# Treatments for Trauma-Induced Coagulopathy: Protocol for a Systematic Review and Meta-Analysis

**DOI:** 10.2196/49582

**Published:** 2023-12-11

**Authors:** Yuki Itagaki, Mineji Hayakawa, Yuki Takahashi, Yuichiro Sakamoto, Shigeki Kushimoto, Yutaka Eguchi, Yoshinobu Seki, Kohji Okamoto

**Affiliations:** 1 Department of Emergency Medicine Hokkaido Univerisity Sapporo Japan; 2 The Scientific and Standardization Committee on DIC of the Japanese Society on Thrombosis and Hemostasis Tokyo Japan; 3 Department of Emergency Medicine Saga Univerisity Saga Japan; 4 Department of Emergency and Critical Care Medicine Tohoku University Sendai Japan; 5 Department of Emergency Medicine Shiga University Shiga Japan; 6 Department of Hematology Uonuma Institute of Community Medicine Niigata University Niigata Japan; 7 Department of Surgery Kitakyushu City Yahata Hospital Kitakyushu Japan

**Keywords:** trauma-induced coagulopathy, coagulation factor, transfusion, blood, clot, clotting, coagulation, coagulopathy, systematic, review methods, review methodology, meta-analysis, meta-analyses, bleeding, trauma, hemorrhage, traumatic, injury

## Abstract

**Background:**

Trauma-induced coagulopathy (TIC) is a common and potentially life-threatening coagulopathy as a result of traumatic injury, characterized by abnormal blood clotting and bleeding. Although several treatments have been proposed for TIC, their effectiveness and safety remain unclear. Further, numerous systematic reviews and meta-analyses on trauma have been conducted; however, to our knowledge, there is no systematic review and meta-analysis that specifically focuses on TIC management. Therefore, a comprehensive synthesis of the available evidence on interventions for TIC is needed.

**Objective:**

This systematic review and meta-analysis aim to evaluate the effectiveness and safety of interventions for the management of TIC.

**Methods:**

We will conduct a systematic review and meta-analysis of randomized and nonrandomized controlled trials as well as observational studies regarding severe trauma in patients with TIC. The interventions will include administration of coagulation factor concentrates, tranexamic acid, and blood component products. The control group will be managed with an ordinal transfusion or administered placebo. The primary outcome will be in-hospital mortality. We will search the electronic databases of MEDLINE (PubMed), Web of Science, and the Cochrane Central Register of Controlled Trials. Two reviewers will independently screen the titles and abstracts, retrieve the full text of the selected articles, and extract essential data. We will apply uniform criteria for evaluating the risk of bias associated with individual randomized controlled trials and nonrandomized trials based on the Cochrane risk-of-bias tool. Risk ratio values will be expressed as point estimates with 95% CIs. Continuous variables will be expressed as the mean difference along with their 95% CIs and *P* values. We will assess the strength of evidence using the GRADE (Grading of Recommendations Assessment, Development, and Evaluation) approach. This review will be the first systematic review and meta-analysis providing information on the effectiveness and safety of interventions for the management of TIC, including the administration of coagulation factor concentrates, tranexamic acid, and blood component products. Ethics approval and patient consent were not required for this study protocol, as we conducted a systematic review and meta-analysis of publicly available data, without any direct involvement of human participants.

**Results:**

We will summarize the selection of the eligible studies using a PRISMA (Preferred Reporting Items for Systematic Reviews and Meta-Analyses) flowchart. The results will be presented in a table summarizing the evidence. The results of the meta-analysis will be depicted using figures and forest plots.

**Conclusions:**

This systematic review will provide updated information on the efficacy and safety of using coagulation factor concentrates, tranexamic acid, and blood component products for patients with TIC. To our knowledge, there is no systematic review and meta-analysis that specifically focuses on treatments for TIC.

**Trial Registration:**

UMIN registry UMIN000050170; https://tinyurl.com/yr8pcrj6

**International Registered Report Identifier (IRRID):**

DERR1-10.2196/49582

## Introduction

Trauma remains a major cause of death globally [1]. Death during the early phase of trauma is primarily attributable to uncontrolled bleeding, which is exacerbated by trauma-induced coagulopathy (TIC) [2-4]. Pathophysiology and clinical aspects of TIC comprise blood loss, consumption of coagulation factors, dilution, and fibrinolytic activation [2-5]. TIC correlates with an increased demand for massive transfusion as well as higher mortality. Thus, effective treatment of TIC is crucial for decreasing deaths following trauma [3-5]. Although the conceptual definition of TIC was proposed by the International Society on Thrombosis and Haemostasis, the clinical definition of TIC has not reached a consensus [3].

Randomized controlled trials (RCTs) have investigated the effectiveness of transfusion, administration of coagulation factor concentrates, and tranexamic acid for hemostasis following severe trauma [5-12]. Several systematic reviews and meta-analyses have also been conducted based on these studies [13-15]. However, the inclusion criteria for these reviews were mostly for trauma with severe bleeding or traumatic brain injury with severe consciousness disturbance, and they were not focused on patients with TIC. We speculate that the effectiveness of interventions for coagulation abnormalities is greatest in patients with TIC.

In addition, the administration of tranexamic acid for severe trauma has been investigated by large RCTs, and it is now globally used for early-stage trauma management with a proven lifesaving benefit [6,8,16]. Furthermore, a meta-analysis has indicated the beneficial effect of tranexamic acid on trauma [15]. Tranexamic acid is a potent antifibrinolytic agent, and its clinical effectiveness demonstrated in previous RCTs is presumably based on this antifibrinolytic effect. Therefore, tranexamic acid administration is likely to be more effective for severe trauma with TIC. However, the inclusion criteria of these RCTs and the meta-analysis were not focused on patients with TIC [6,8,15].

To our knowledge, there is no systematic review and meta-analysis specifically focusing on treatments for TIC. From late 2010 to 2020, RCTs investigating the efficacy of coagulation factor concentrates for TIC were published [5,11,12,17]. Due to the small number of RCTs and the biased setting of open-label interventions in these RCTs, observational studies continue to provide novel and helpful insights [18-20]. Therefore, a comprehensive synthesis of the available evidence on interventions for the management of TIC is needed. This is the first systematic review and meta-analysis aimed at evaluating the effectiveness and safety of interventions for TIC, including the administration of coagulation factor concentrates, tranexamic acid, and blood component products.

## Methods

### Protocol Registration

The study protocol was registered in the University Hospital Medical Information Network (UMIN000057146). This systematic review and meta-analysis will be reported according to the PRISMA (Preferred Reporting Items for Systematic Reviews and Meta-Analyses Protocols) guidelines.[21]

### Database Search

To retrieve relevant articles, we will perform a literature search of the following electronic bibliographic databases: MEDLINE (PubMed), Web of Science, and the Cochrane Central Register of Controlled Trials. We will examine the references cited in relevant articles to ascertain whether additional studies can be found. The details of the search strategy are available in Multimedia Appendices 1-3.

### Study Types

We will include controlled trials (including RCTs and other controlled trials) and observational studies published until December 17, 2022. Studies will be excluded if they do not clearly report the population, treatment, or outcomes of interest. Animal studies will also be excluded. Grey literature, such as conference proceedings and abstracts, will be included. If 2 or more studies are published using the same or overlapping cohorts, the one with the most recent or larger cohort will be included. Only articles published in English will be included.

### Study Population

We will include patients with severe trauma and TIC admitted to the hospital. However, there are no international diagnostic criteria for TIC. Therefore, we will include patients with some abnormalities in coagulation or fibrinolysis upon admission to the hospital as patients with TIC and collect the definition of abnormality in coagulation or fibrinolysis in each study.

### Intervention and Control

Intervention types will include the supplementation of coagulation factor concentrations, such as prothrombin complex concentrate (PCC), fibrinogen concentrate, tranexamic acid, and blood component products during the acute phase of trauma. The control group will be managed with an ordinal transfusion or administered placebo. We will not restrict our review to the product type and amount or the timing of the administration of coagulation factors.

### Outcomes

The primary outcomes will be all-cause mortality and the quantity of transfusion. The secondary outcomes will be thrombotic events (ie, deep venous thromboses, pulmonary embolism, myocardial infarctions, and strokes), length of intensive care unit stay, and hospital stay.

### Study Selection

Citations will be stored, and duplicates will be removed using EndNote Software (Thomson Reuters). Rayyan software will also be used for the systematic review process [22]. The titles and abstracts of studies retrieved using the search strategy will be screened independently by 2 reviewers (YI and YT) to identify studies that potentially meet the inclusion criteria. The full texts of these potentially eligible studies will be retrieved and assessed independently by 2 reviewers (YI and YT). Any disagreement about the eligibility of studies will be resolved through consultation with a third reviewer (MH).

### Data Extraction

Using a standardized prepiloted form, data from the included studies will be extracted to assess the quality of the studies and methods of data synthesis. The extracted information will include data on the following variables: study setting, study population, baseline characteristics of the participants, details of the interventions and control conditions, study methodologies, outcomes, and assessments of the risks of bias. Two independent reviewers (YI and YT) will independently extract the data, and discrepancies will be resolved through discussion with a third author (MH). Missing data will be obtained through requests from the authors.

### Assessment of Risk of Bias in Individual Studies

Two independent reviewers (YI and YT) will assess risk of bias in individual studies as well as the methodological quality of the articles, and disagreements will be resolved by discussion with a third reviewer (MH). We will apply uniform criteria for evaluating the risk of bias associated with individual RCTs based on the tool for assessing risk of bias in randomized trials [23]. Each study will be assessed for (1) random sequence generation, (2) allocation concealment, (3) blinding of participants and personnel, (4) blinding of related outcome assessment, (5) incomplete outcome data, (6) selective reporting, and (7) other bias. We will also use the “Risk Of Bias In Non-randomised Studies – of Interventions” tool for individual nonrandomized studies [24].

### Data Summary

We will perform a meta-analysis when data are available in 1 or more trials according to the Cochrane Handbook for Systematic Reviews of Interventions. For binary variables, values for the risk ratio or odds ratio will be expressed as a point estimate with 95% CIs. Continuous variables, such as the length of intensive care unit stay, will be expressed as their mean difference with 95% CIs and *P* values. If quantitative synthesis is not appropriate for a particular outcome, we will provide a qualitative summary.

### Data Synthesis

We will provide estimates of the findings from the included studies according to a random effects model. A random effects model incorporates statistical heterogeneity and provides a more conservative estimate of the pooled effect size compared to a fixed effects model. We will not perform any multiple imputations for missing data, data synthesis, or analysis of randomized trials. All statistical analyses, including risk of bias within studies or across studies, will be performed using Review Manager (RevMan; version 5.4; Cochrane Collaboration 2019) [25]. The level of statistical significance will be set at *P*<.05.

### Assessment of Heterogeneity

Statistical heterogeneity will be assessed using the Mantel-Haenszel *χ^2^* test and *I*^2^ (*I*^2^>50%=significant heterogeneity) [26]. The presence of clinical heterogeneity will be considered in the decision to conduct a quantitative synthesis of data or perform sensitivity analyses.

### Sensitivity Analysis

We will examine the robustness of this meta-analysis by conducting a sensitivity analysis according to the different components of the Cochrane risk of bias tool. We will also perform sensitivity analyses by excluding studies in which the overall risk of bias is high or unclear.

### Subgroup Analysis

Subgroup analyses will be performed according to the type of coagulation factors, type of trauma (traumatic brain injury will be separately investigated), timing of administration, or type of coagulopathy by each clinical criterion.

### Assessment of Reporting Bias

To assess publication bias, we will create funnel plots for mortality.

### Rating the Strength of Evidence Using the GRADE Approach

Two authors (YI and YT) will assess the strength of evidence independently using the GRADE (Grading of Recommendations Assessment, Development and Evaluation) approach [27]. The quality of evidence will be assessed for each outcome and categorized as high, moderate, low, or very low according to the GRADE approach.

### Patient and Public Involvement and Study Dissemination

Patients and the public were not involved in the design of this protocol. Our findings will be presented at relevant scientific conferences and disseminated through publication in peer-reviewed journals.

### Ethical Considerations

Approval from an ethics committee was not required because in this study, we conducted a systematic review and meta-analysis of publicly available data, without any direct involvement of human participants.

## Results

We will summarize the selection of eligible studies using a PRISMA flowchart. The results will be presented in a table summarizing the evidence. The results of the meta-analysis will be depicted using figures and forest plots.

## Discussion

The use of a coagulation factor concentrate enables a rapid and strong supply of coagulation factors and is recommended in patients with severe trauma in recent international guidelines [28]. Recently, RCTs on the use of fibrinogen concentrates in patients experiencing trauma have increased [5,10-12,29,30]. As per a meta-analysis conducted in 2020 [14], fibrinogen concentrates do not decrease the mortality rate. However, the inclusion criteria in this study did not focus on patients with TIC [14]. A meta-analysis of PCC administration focused on patients with Prothrombin Time-International Normalized Ratio >1.5 was reported in 2021 [13]. In this study, PCC was shown to reduce the mortality rate compared with fresh frozen plasma alone. Although platelets play a fundamental role in hemostasis and have a close counteraction with coagulation factors [2], the effectiveness of early supplementation of platelets for trauma is poorly investigated [31]. Tranexamic acid is now proven to be beneficial for life-threatening conditions [6,8,16] and is recommended by European guidelines [32] with high-quality trials. However, the studies mentioned above were not focused on patients with TIC. TIC is present in up to 25%-35% of patients experiencing trauma upon admission to the emergency department and is associated with a poor prognosis [4,33-35]. Therefore, we speculate that the intervention’s effectiveness in addressing coagulation and fibrinolysis abnormalities is greatest in patients with TIC, and a systematic review and meta-analysis focused on the management of patients with TIC is needed.

This study will be the first endeavour to undertake a systematic review and meta-analysis on the effectiveness of early interventions for the management of TIC, involving the provision of coagulation factors and the administration of tranexamic acid. Patients with TIC, who represent a substantial subpopulation with severe and high mortality rates, require special attention [2]. Determining the effectiveness of treatments for this cohort of patients would yield significant advantages. In addition, further studies on interventions that influence the coagulation-fibrinolytic system, such as the use of coagulation factor concentrates and tranexamic acid, may offer more precise insights into their effectiveness in patients with TIC, as opposed to those without TIC. Additionally, this study may facilitate further research focused on assessing the diagnostic measures of TIC and thresholds of abnormal lab data for defining TIC by collating existing studies. As such, our findings will contribute to the current knowledge on the treatment of TIC.

## Appendix

Multimedia Appendix 1 Cochrane Central Register search strategy.

Multimedia Appendix 2 MEDLINE (via PubMed) search strategy.

Multimedia Appendix 3 Web of Science search strategy.

## Abbreviations

GRADE: Grading of Recommendations Assessment, Development and Evaluation
PCC: prothrombin complex concentrate
PRISMA: Preferred Reporting Items for Systematic Reviews and Meta-Analyses Protocols
RCT: randomized controlled trial
TIC: trauma-induced coagulopathy
