# Supplementary material for: Treatments for Trauma-Induced Coagulopathy: Protocol for a Systematic Review and Meta-Analysis
Source: JMIR Res Protoc. 2023 Dec 11;12:e49582. doi: 10.2196/49582 (PMC10750238; doi:10.2196/49582)
Supplement: Multimedia Appendix 1 [file resprot_v12i1e49582_app1.docx]

**Appendix : CENTRAL search strategy**

#1 trauma[mh]

#2 coagulopathy[tiab]

#3 tranexamic acid[tiab]

#4 blood coagulation factor[tiab]

#5 platelet[tiab]

#6 blood component transfusion[tiab]

#7 prothrombin complex[tiab]

#8 #1 AND #2

#9 #3 OR #4 OR #5 OR #6 OR #7

#10 #8 AND #9
