# Supplementary material for: Treatments for Trauma-Induced Coagulopathy: Protocol for a Systematic Review and Meta-Analysis
Source: JMIR Res Protoc. 2023 Dec 11;12:e49582. doi: 10.2196/49582 (PMC10750238; doi:10.2196/49582)
Supplement: Multimedia Appendix 2 [file resprot_v12i1e49582_app2.docx]

**Appendix : MEDLINE(via PubMed) search strategy**

#1 injury[mh]

#2 trauma[tiab]

#3 Blood **Coagulation Disorders [mh]**

**#4 coagulopathy[tiab]**

#5 #1 OR #2

#6 #3 OR #4

#7 Blood Coagulation Factors[mh]

#8 **Tranexamic Acid[mh]**

**#9 platelet[mh]**

**#10 blood component transfusion[mh]**

#11 prothrombin complex[tiab]

#12 #5 AND #6

#13 #7 OR #8 OR #9 OR #10 OR #11

#14 #12 AND #13
