# Supplementary material for: Treatments for Trauma-Induced Coagulopathy: Protocol for a Systematic Review and Meta-Analysis
Source: JMIR Res Protoc. 2023 Dec 11;12:e49582. doi: 10.2196/49582 (PMC10750238; doi:10.2196/49582)
Supplement: Multimedia Appendix 3 [file resprot_v12i1e49582_app3.docx]

**Appendix : Web of science search strategy**

#1 trauma

#2 coagulopathy

#3 tranexamic acid

#4 blood coagulation factor

#5 platelet

#6 blood component transfusion

#7 prothrombin complex

#8 #1 AND #2

#9 #3 OR #4 OR #5 OR #6 OR #7

#10 #8 AND #9
